# Correcting for cell-type composition bias in epigenome-wide association studies

**DOI:** 10.1186/gm540

**Published:** 2014-03-25

**Authors:** Robert Lowe, Vardhman K Rakyan

**Affiliations:** 1 The Blizard Institute, Barts and The London School of Medicine and Dentistry, Queen Mary University of London, London, E1 2AT, UK

## Abstract

Recent epigenome-wide association studies have indicated a potential role for epigenetic variation in the etiology of complex human diseases. However, one major challenge is to distinguish true epigenetic variation from changes caused by differences in cellular composition between the disease and non-disease state, a problem that is particularly relevant when analyzing whole blood. For studies with large numbers of samples, it can be expensive and very time consuming to perform cell sorting, and it is often not clear which is the correct cell type to profile. Two recently published papers have attempted to address this confounding issue using bioinformatics.

## Cell-type composition as a confounding factor in epigenome-wide association studies

Despite the success of genome-wide association studies (GWASs) in identifying common disease-associated loci in humans, a substantial proportion of disease causality remains unexplained. Consequently, there is now strong interest in exploring the role of inter-individual epigenetic variation in disease pathogenesis. Epigenome-wide association studies (EWASs) have been initiated by many different groups to systematically catalogue epigenetic variation (with an emphasis on DNA methylation) in various diseases. These EWASs have the potential to yield important new insights into disease pathogenesis and to provide biomarkers, but conducting such studies presents challenges not encountered in GWASs [1]. The main challenge is that whereas germline genetic variation is present and unaltered in virtually every cell of a given individual, epigenetic profiles are subject to temporal, spatial and developmental dynamics, and are influenced by environmental factors. One key issue in EWASs is therefore the cell type to profile, as only one or a few cell types may have an etiological role in the disease. Often cells from the target tissue are not easily available in large enough numbers to provide adequate statistical power, and thus surrogate tissues, most commonly whole blood, are used instead. The expectation is that the surrogate tissue will reflect epigenomic perturbations found in the target tissue, or at least yield biomarkers that - although not directly causative of the disease - can still be used for predictive, diagnostic or prognostic purposes.

Regardless of whether target or surrogate tissues are used, a major issue for both designing and correctly interpreting an EWAS is to determine whether disease-associated variation is truly epigenetic, or is the result of differences in cellular composition between the disease and non-disease state. For example, during aging the cellular composition of blood is often altered [2], and thus the measured epigenetic variation may be due to differences in tissue-specific profiles between different blood subsets. The ideal solution to this problem is to isolate and profile individual cell subsets. In many cases this is not practicable on a large scale and consequently there is a reliance on unsorted tissues. Two recent papers - by Zou *et al.*[3] and Jaffe and Irizarry [4] - highlight this issue, and propose a *post hoc* bioinformatics solution to correct for confounding cell-type bias in EWASs.

## Accounting for cellular heterogeneity is critical in epigenome-wide association studies

Writing in *Genome Biology*, Jaffe and Irizarry [4] present a method for accounting for cellular heterogeneity in whole blood using an existing reference database of sorted blood cells (granulocytes, CD8^+^ and CD4^+^ T cells, CD56^+^ natural killer cells, CD19^+^ B cells and CD14^+^ monocytes) from six adult male samples. They found that a striking 63.5% of CpG sites showed differences in methylation across the cell types, and provide a statistical summary of cell-type variability as an additional file. The method they report is based on Houseman *et al*. [5], which uses a random effects model at each of the CpGs, but they have adjusted it somewhat for genetics by removing probes containing an annotated single nucleotide polymorphism (SNP) at the CpG site of interest. The algorithm used is now freely available in the popular minfi Bioconductor package [6], which will allow researchers to incorporate it into existing pipelines.

## Epigenome-wide association studies without the need for cell-type composition

Zou *et al*. [3] report a non-reference-based method for correcting cell-type composition that differs between cases and controls. This approach has a benefit over that of Jaffe and Irizarry in that it can be applied to any tissue type rather than just blood. The method is an adjustment of an existing algorithm called FaST-LMM, which has previously been used in a GWAS [7] and performs a linear mixed model analysis. Unfortunately, use of the technique is restricted to specific case versus control studies. This means it is not possible to perform regression analysis or analysis with multiple conditions, and it also restricts the power of the method in twin design studies, as there is no opportunity to perform pair-wise analysis. Liu *et al.*[8] recently reported an EWAS for rheumatoid arthritis in which they corrected for cellular heterogeneity using a reference-based approach; in their paper, Zou *et al.* showed that both methods produce consistent results. They also applied their approach to breast cancer data from The Cancer Genome Atlas (TCGA) and produced results that showed enrichment for known genes and pathways. One concern regarding the method is that its power relies on a small number of loci being true associations (see Supplementary Figure 8 of [3]); that is to say, the number of differences due to the phenotype of interest is small (<1% of sites tested). This is a potential concern when investigating cancer datasets, for example, in which a large number of changes occur.

## Problems with correcting for cellular heterogeneity

The importance of these new methods is that they may overcome the problem of spurious correlations related to differences in cell populations. The finding by Jaffe and Irizarry that up to 63.5% of CpGs show significant differences in methylation in blood cell populations would mean that some variation in the phenotype of interest may occur at these sites by chance. Indeed, they applied their method to measure cell composition in peripheral blood taken from a number of studies that looked into age-related methylation differences (aDMPs). They found that 86.7% of aDMPs varied significantly (*P* value <0.05) across cell type. It has been reported [9], however, that a reasonable proportion of these aDMPs are shared among tissues and hence it would be unlikely that the cause of these differences in blood is the difference in cell-type composition, despite being labelled as such. It is also possible that some, or even many, disease-specific epigenetic changes occur at tissue-specific sites [10]. Therefore, although these methods provide useful insights they do not provide a holy grail for analysis, and any findings from EWASs must be considered very carefully.

## Abbreviations

aDMP: Age-related methylation difference; EWAS: Epigenome-wide association study; GWAS: Genome-wide association study; SNP: Single nucleotide polymorphism; TCGA: The Cancer Genome Atlas

## Competing interests

The authors declare that they have no competing interests.
